# Inflatable penile prosthesis in the radical prostatectomy patient: a review

**DOI:** 10.12688/f1000research.15241.1

**Published:** 2018-06-19

**Authors:** Nelson Bennett, I-shen Huang

**Affiliations:** 1 Department of Urology, Northwestern Memorial Hospital, Chicago, IL, 60611, USA; 2 Department of Urology, Taipei Veterans General Hospital, Taipei, Taiwan

**Keywords:** erectile dysfunction, penile prosthesis, prostate surgery

## Abstract

In the population of patients with prostate cancer, survivorship has come to the forefront of continuity-of-care. In addition to urinary control, erectile function is a significant issue after radical pelvic surgery. Penile prosthesis surgery remains an excellent option for restoring erectile function to those for whom more conservative measures have failed. This review article outlines the anatomical, surgical and post-operative consideration involved in the placement of a penile prosthesis in this special patient population.

## Erectile dysfunction (ED) rates after radical prostatectomy (RP)

Penile erection is the culmination of complex series of highly integrated phenomena involving the central nervous system, peripheral nervous system, endocrine system and the vascular system. These systems must be working in concert and at a high level in order for full erection to occur. ED may occur when there is an impairment or derangement to any of these systems. ED has been defined as the inability of a man to achieve or maintain an erection sufficient for satisfactory penetrative sexual intercourse ^1^.

Post-RP ED may be neurogenic, venogenic, arteriogenic or a combination of these etiologies. In all cases, injury of the cavernous nerves occurs during dissection of the prostate. The injury, whether caused by contact, traction, electro-cautery or transection, initiates a cascade of events that culminates in ED. Microscopically, cavernous nerve-fiber injury initiates Wallerian degeneration that will incapacitate the axon back to the cell body, typically at the level of the spinal cord. The lack of nervous input at the end-organ (cavernous muscle) is a major contributor to cavernosal tissue degeneration and atrophy ^2, 3^. Venous leakage is another underlying mechanism responsible for ED after prostatectomy. Bilateral cavernosal nerve injury has been shown to induce cavernosal smooth muscle death which will lead to veno-occlusive dysfunction ^3^. Chronic hypoxia, denervation, and activation of the TGF-β cascade are believed to initiate the apoptosis process, and increase the deposition of collagen-laden scar tissue ^4– 7^.

The initiating factor in the development of arteriogenic ED as a result of radical pelvic surgery is transection of the accessory pudendal arteries. These arteries arise from the peri-prostatic vasculature and course toward the penis and providing a significant portion of the arterial inflow required for normal erectile function ^8, 9^.

The incidence of post-prostatectomy ED has been reported to be between 29-88% ^10– 16^. This wide range of values can largely be attributed to failure to control various confounding factors including age, degree of nerve sparing, different definition of potency and preoperative ED. The CaPSURE study revealed that only 20% of patients returned to their preoperative baseline potency levels 1 year after RP ^12^. In the Prostate Cancer Outcome Study (PCOS), 59.9% of men self-reported having ED following RP ^14^. Similar results were found by the Memorial Sloan-Kettering Cancer Center group ^17– 19^. According to The Scandinavian Prostate Cancer Group, men who chose watchful waiting had a 45% incidence of ED. Those that selected RP as a treatment option experienced an incidence of ED at 80% ^20^.

## Inflatable penile prosthesis (IPP) utilization after RP

Phosphodiesterase-5 inhibitors (PDE5Is) initially were the first-line treatment for ED secondary to RP. However, they are not uniformly effective. In a subset of patients who initially respond, the response deteriorates over time as progressive cavernosal tissue damage occurs and subsequent venous leak develops ^18, 21, 22^. Penile implant surgery is a viable treatment option in patients in whom nonsurgical ED treatments are unsatisfactory or are associated with adverse effects ^23^. The utilization rate of penile implants after RP varies from 0.8 to 1.9% ^24, 25^. These reported rates have been obtained from analysis of the Surveillance Epidemiology and End Results (SEER) cancer registry database. The higher rate of 1.9% in a study by Stephenson *et al.* ^25^ is probably due to a younger cohort, as 45% of subjects were younger than 65 years. There are numerous reasons that could be postulated for low utilization of penile implants. Firstly, prostate cancer treatment modalities have improved, thereby decreasing the incidence of ED that is unresponsive to nonsurgical intervention. Secondly, effective nonsurgical treatment modalities have been developed as alternatives to surgical treatment, predominantly PDE5Is. Meta-analysis of contemporary publications by Tal *et al.* revealed an overall erectile function recovery rate of 58% among men younger than 60 years after RP ^26^. The study acknowledged that the definitions of ED were different in each member study and some men used of PDE5i’s for erectile rigidity ^26^. Nevertheless, Stanley *et al.* found that there was no significant change in total number of penile implant procedures over a 10-year period before and after the introduction of sildenafil citrate ^27^.

## Surgical considerations

### Cylinder placement

***Technique of dilation of fibrotic corpora.*** Corporal crossover and urethral perforation are more likely to occur during dilation of fibrotic corpora ^28^. After placing the stretched penis into anatomical position (urethral meatus pointing in a superior direction), a small dilating instrument is placed into the corporotomy and slowly advanced in a latero-superior direction until it reaches the mid-glans penis. Sequential dilation is needed until the cavernosa accepts a 11-12 Fr. dilator. The most important caveat to remember is to orient the dilating instrument in a latero-superior direction when advancing the dilator within the corporal space ^28^. This will prevent corporal crossover, as well as, provide a visual representation of the location of the dilator within the corpora. Tools used for dilation may include Metzenbaum scissors, Hagar dilators, Brooks dilators, Rossello cavernotomes, Mooreville cavernotomes, and the dialmezinsert dilator ^29^.

***Technique of corporal measurement.*** After corporotomy, PDS 2-0 stay sutures of are placed at the corporotomy edge. The sutures are used for traction, as well as, for closure of the corporotomy after insertion of the prosthesis. Historically, corporal dilation would then ensue with Brooks or Hegar dilators. After ensuring the corporal space was dilated to 14 mm, the corporal length was then measured and the prosthesis placed. In the contemporary setting, many implanters first measure the length of the corpora with the Furlow ^30^. This narrow device provides enough passive dilation to place an inflatable prosthesis, especially if the corpora are non-fibrotic. If the corpora are fibrotic, the implanter would then dilate the corpora to ensure smooth insertion of the prosthesis ^31^. When dilating or measuring the corpora, it is imperative to direct any instrumentation laterally to avoid urethral injury or corporal crossover ^32^.

To measure the length of the corpora, gently advance the cylindrical measuring device proximally within the corporal space. When the bottom of the corpora cavernosa is reached, a measurement is recorded. Next, the penis is placed and securely held in “anatomical” position. The measuring instrument is passed distally towards the glans penis while angling the instrument laterally. A distal measurement is recorded and added to the proximal measurement. There should be no more than a 1-cm discrepancy between the right and left corpora. A >1 cm difference suggests incomplete dilation, corporal crossover, crural perforation or urethral perforation.

### Prevention and management of corporal crossover

Both proximal and distal corporal crossover can happen during dilation, measurement, or cylinder placement ^33^. In addition, the initial correct distal tunneling technique using laterally directed dilators will help avoid crossover ^28^. Side-by-side placement of the Brooks or Hagar dilators in each corpus to check for symmetry and proper positioning is the best way to check for proximal or distal crossover ^34^. If a crossover is detected, the dilator may simply be redirected with the contralateral dilator left in place to prevent repeat crossover ^28^.

### Prevention and management of perforation

Proximal crural perforation is suspected when there is asymmetry of proximally positioned dilators or a significant length differential. Gentle dilation and corporal measurement can prevent this manageable complication. In the event of proximal perforation, management may take the form of direct repair of perforation, creation and placement of windsock using an “off-the-shelf” implantable graft, creation of a hammock using a rear-tip-extender, or anchoring of cylinder tubing to tunica ^35, 36^. A simple ‘U-type’ suture will prevent proximal cylinder migration.

Distal corporal and urethral perforation requires termination of the procedure, especially if distal perforation occurs during dilation of the first side ^37^. If a second side is perforated after successful cylinder placement of the contralateral side, the single cylinder may be left on the non-perforated side ^38^. Urethral tear may be repaired or, if very small, left to heal over the catheter ^39^. Many surgeons will abandon the case during urethral injury in fear of prosthesis infection.

## Reservoir placement

The reservoir is normally placed in space of Retzius. This is done to reduce the creation of inguinal floor weakness and to reduce the potential risk of visceral injury. After ensuring complete bladder drainage, the index finger is placed through the IPP incision and advanced to the medial aspect of the external inguinal ring. Using firm pressure, the finger is advanced in a posterior direction, piercing transversalis fascia. If finger pressure is inadequate, the fascia can be perforated with the tip of an instrument (scissors or clamp). This action will create a rent large enough to insinuate an index finger into the space of Retzius. Alternatively, a long-bladed nasal speculum is useful in expanding the retroperitoneal space.

If the space of Retzius is obliterated due to previous pelvic surgery, ectopic placement of the reservoir should be considered ^40, 41^. The reservoir may be placed in the deep to the abdominal musculature superior or posterior to transversalis fascia ^42^. A Foerster or Debakey clamp may be used to advance the deflated reservoir to its ectopic position. Stember *et al* reported the outcomes of 2687 men who underwent ectopic reservoir placement ^42^. In total, 83%, of men had reservoirs placed posterior to the transversalis fascia. The remainder had reservoirs placed in the anterior transversalis space. No injuries to the bowel or major blood vessels occurred with initial insertion of the reservoir, however two patients experienced bladder injury. Eight patients required reservoir revision secondary to herniation ^42^.

### Prevention and management of bladder and bowel perforation

The most serious intraoperative complications of penile prosthesis insertion occur during reservoir placement ^43^. Traditionally, the reservoir is placed blindly in a retrograde fashion into the space of Retzius through a penoscrotal incision. The serious potential complications include vascular injury, bowel perforation and bladder perforation ^44^.

Vascular injury (arterial or venous avulsion) may occur during overly aggressive finger or instrument dilation of the inguinal ring. In the event of brisk bleeding, tapenade with an index finger or sponge stick is advised ^45^. Direct access into the space of Retzius is then accomplished through an inguinal incision. Meticulous inspection of the pelvic sidewall will frequently localize the avulsed venous vessel. In the event of vascular injury of the major pelvic vessels, consultation from a vascular surgeon is recommended.

Bladder injury is a complication that should be recognized and managed immediately. Prior pelvic surgery or radiation may result in adherence or fixation to the pelvic sidewall. Bladder perforation can happen while piercing the transversalis fascia ^46^. Emptying the bladder prior to placing the reservoir can decrease the incidence of these injuries. Bladder injury is noted when gross blood is seen in the urine or the observation of urine emanating through the IPP incision ^31^. The injury can be confirmed via flexible cystoscopy or by an on-table cystogram. In case of bladder injury due to scissors, the reservoir should be removed and placed on the contralateral side. The bladder should be drained for 7–10 days. Cystogram should be done prior to catheter removal.

The bowel may be damaged in a similar mechanism to bladder injury during reservoir placement ^31^. Upon recognition bowel injury (succus entericus in the wound), a general surgeon should be consulted for repair and the prosthesis removed.

## Satisfaction rates after penile prosthesis implantation

Serial reports regarding of penile prosthesis surgery outcomes demonstrate excellent long-term mechanical reliability of contemporary prosthesis models; satisfaction is superior when compared to PDE5Is and injections. Carson *et al.* performed a retrospective long-term multicenter study on 372 patients who underwent penile prosthesis implant and focused on the longevity, morbidity and patient satisfaction ^47^. More than 80% of patients were satisfied with the function of the device, the ease of inflation, and level of rigidity. Steege *et al.* reported that patient satisfaction with semi-rigid prostheses was higher than 90%, however, inflatable devices enjoy a slightly higher satisfaction ^48^. Holloway and Farah reported that the AMS 700 Ultrex prosthesis had a 86% patient and 76% partner satisfaction at a mean postoperative follow-up of 42 months ^49^. In a study by Rajpurkar *et al*., the investigators demonstrated significantly enhanced erectile function and sexual satisfaction when compared to those receiving sildenafil and intracavernosal prostaglandin ^50^.

The psychosexual adaptation to penile implant may take up to 6 months. The patients experience a marked enhancement in erectile function with elevation of libido. Apprehension regarding the maintenance of an erection during intercourse is markedly assuaged. In addition to an upsurge in the regularity of sexual activity, a decrease in feeling of sadness, depression, anxiety and an improvement in sexual satisfaction has also been noted ^51^.

Two major factors contributing to high level of satisfaction are rapid generation of erection and consistently excellent rigidity. Other factors, such as degree of postoperative pain and swelling, postoperative complications, ease of concealment, cosmetic outcome, device function, ease of use and partner acceptance, are critical in determining the patient satisfaction. Potential predictors of patient dissatisfaction with penile prosthesis include Peyronie’s disease, a body mass index >30, or previous RP ^52^.

## Predictors of requiring a penile prosthesis

The natural recovery time of erectile function may be up to 24 months after radical pelvic surgery; however, resultant penile rigidity may be maximized by early treatment with intracorporal injection therapy ^53– 55^. Various different symptomatic treatments are available for patients who fail to regain a natural erection. Sildenafil becomes effective in the late recovery phase as the nerves recover from intraoperative injury ^54^. At 2 or more years from surgery, the recovery of natural function and improved response from other therapies is unlikely and implantation of penile prosthesis is warranted ^54^. A study by Tal *et al.* found that men who had surgery as an initial treatment (versus radiotherapy), were of a younger age, were of African/American/Hispanic race, were unmarried, and were living in a geographic region other than the North-east were more likely to utilize penile implants ^25^. Similarly, in a retrospective analysis of claims data from Medicare & Commercial databases of 3928 men undergoing penile prosthesis, Segal and Burnett elucidated the factors with the greatest predictive strength of penile prosthesis implantation, which included a diagnosis of prostate cancer, a diagnosis of diabetes mellitus and previous treatment with first-line ED therapy ^56^.

## Conclusion

In the population of patients with prostate cancer, the concept of survivorship has become a central tenet of patient care. To that end, quality of sexual life, and especially erectile function has become a significant issue. Penile prosthesis surgery remains an excellent option for restoring erectile function to those who fail more conservative measures. IPP implantation enjoys high satisfaction rates for patients and their partners. Intraoperative complications can be distressing, but with prompt recognition, most of these complications can be navigated with excellent postoperative results.

## Data availability

No data are associated with this article.

## References

[1] NIH Consensus Conference. Impotence. NIH Consensus Development Panel on Impotence. *JAMA.* 1993;270(1):83–90. 10.1001/jama.1993.03510010089036 8510302

[2] Küry P Stoll G Müller HW: Molecular mechanisms of cellular interactions in peripheral nerve regeneration. *Curr Opin Neurol.* 2001;14(5):635–9. 10.1097/00019052-200110000-00013 11562576

[3] User HM Hairston JH Zelner DJ: Penile weight and cell subtype specific changes in a post-radical prostatectomy model of erectile dysfunction. *J Urol.* 2003;169(3):1175–9. 10.1097/01.ju.0000048974.47461.50 12576876

[4] Klein LT Miller MI Buttyan R: Apoptosis in the rat penis after penile denervation. *J Urol.* 1997;158(2):626–30. 10.1016/S0022-5347(01)64572-5 9224381

[5] Yamanaka M Shirai M Shiina H: Loss of anti-apoptotic genes in aging rat crura. *J Urol.* 2002;168(5):2296–300. 10.1016/S0022-5347(05)64374-1 12394778

[6] Yao KS Clayton M O'Dwyer PJ: Apoptosis in human adenocarcinoma HT29 cells induced by exposure to hypoxia. *J Natl Cancer Inst.* 1995;87(2):117–22. 7707382 10.1093/jnci/87.2.117

[7] Zhou F Li GY Gao ZZ: The TGF-β1/Smad/CTGF pathway and corpus cavernosum fibrous-muscular alterations in rats with streptozotocin-induced diabetes. *J Androl.* 2012;33(4):651–9. 10.2164/jandrol.111.014456 22016353

[8] Aboseif S Shinohara K Breza J: Role of penile vascular injury in erectile dysfunction after radical prostatectomy. *Br J Urol.* 1994;73(1):75–82. 10.1111/j.1464-410X.1994.tb07460.x 8298902

[9] Droupy S Hessel A Benoît G: Assessment of the functional role of accessory pudendal arteries in erection by transrectal color Doppler ultrasound. *J Urol.* 1999;162(6):1987–91. 10.1016/S0022-5347(05)68084-6 10569553

[10] Catalona WJ Bigg SW: Nerve-sparing radical prostatectomy: evaluation of results after 250 patients. *J Urol.* 1990;143(3):538–43; discussion 544. 10.1016/S0022-5347(17)40013-9 2304166

[11] Kao TC Cruess DF Garner D: Multicenter patient self-reporting questionnaire on impotence, incontinence and stricture after radical prostatectomy. *J Urol.* 2000;163(3):858–64. 10.1016/S0022-5347(05)67819-6 10687992

[12] Litwin MS Flanders SC Pasta DJ: Sexual function and bother after radical prostatectomy or radiation for prostate cancer: multivariate quality-of-life analysis from CaPSURE. Cancer of the Prostate Strategic Urologic Research Endeavor. *Urology.* 1999;54(3):503–8. 10.1016/S0090-4295(99)00172-7 10475362

[13] Schover LR: Sexual rehabilitation after treatment for prostate cancer. *Cancer.* 1993;71(3 Suppl):1024–30. 8428325 10.1002/1097-0142(19930201)71:3+<1024::aid-cncr2820711421>3.0.co;2-2

[14] Stanford JL Feng Z Hamilton AS: Urinary and sexual function after radical prostatectomy for clinically localized prostate cancer: the Prostate Cancer Outcomes Study. *JAMA.* 2000;283(3):354–60. 10.1001/jama.283.3.354 10647798

[15] Walsh PC: Patient-reported impotence and incontinence after nerve-sparing radical prostatectomy. *J Urol.* 1998;159(1):308–9. 9400499

[16] Walsh PC Schlegel PN: Radical pelvic surgery with preservation of sexual function. *Ann Surg.* 1988;208(4):391–400. 10.1097/00000658-198810000-00001 3178328 PMC1493736

[17] Mazzola C Mulhall JP: Penile rehabilitation after prostate cancer treatment: outcomes and practical algorithm. *Urol Clin North Am.* 2011;38(2):105–18. 10.1016/j.ucl.2011.03.002 21621077

[18] Mulhall JP Slovick R Hotaling J: Erectile dysfunction after radical prostatectomy: hemodynamic profiles and their correlation with the recovery of erectile function. *J Urol.* 2002;167(3):1371–5. 10.1016/S0022-5347(05)65303-7 11832735

[19] Nelson CJ Scardino PT Eastham JA: Back to baseline: erectile function recovery after radical prostatectomy from the patients' perspective. *J Sex Med.* 2013;10(6):1636–43. 10.1111/jsm.12135 23551767 PMC4742368

[20] Holmberg L Bill-Axelson A Steineck G: Results from the Scandinavian Prostate Cancer Group Trial Number 4: a randomized controlled trial of radical prostatectomy versus watchful waiting. *J Natl Cancer Inst Monogr.* 2012;2012(45):230–3. 10.1093/jncimonographs/lgs025 23271778 PMC3540876

[21] Ohebshalom M Parker M Guhring P: The efficacy of sildenafil citrate following radiation therapy for prostate cancer: temporal considerations. *J Urol.* 2005;174(1):258–62; discussion 262. 10.1097/01.ju.0000164286.47518.1e 15947650

[22] Teloken PE Parker M Mohideen N: Predictors of response to sildenafil citrate following radiation therapy for prostate cancer. *J Sex Med.* 2009;6(4):1135–40. 10.1111/j.1743-6109.2008.01170.x 19210713

[23] Mulcahy JJ: Erectile function after radical prostatectomy. *Semin Urol Oncol.* 2000;18(1):71–5. 10719936

[24] Stephenson RA Mori M Hsieh YC: Treatment of erectile dysfunction following therapy for clinically localized prostate cancer: patient reported use and outcomes from the Surveillance, Epidemiology, and End Results Prostate Cancer Outcomes Study. *J Urol.* 2005;174(2):646–50; discussion 650. 10.1097/01.ju.0000165342.85300.14 16006930

[25] Tal R Jacks LM Elkin E: Penile implant utilization following treatment for prostate cancer: analysis of the SEER-Medicare database. *J Sex Med.* 2011;8(6):1797–804. 10.1111/j.1743-6109.2011.02240.x 21426495

[26] Tal R Alphs HH Krebs P: Erectile function recovery rate after radical prostatectomy: a meta-analysis. *J Sex Med.* 2009;6(9):2538–46. 10.1111/j.1743-6109.2009.01351.x 19515209 PMC4097184

[27] Stanley GE Bivalacqua TJ Hellstrom WJ: Penile prosthetic trends in the era of effective oral erectogenic agents. *South Med J.* 2000;93(12):1153–6. 11142447

[28] Karpman E: Management of Distal & Proximal Penile Prosthesis Crossover. *J Sex Med.* 2016;13(6):1008–12. 10.1016/j.jsxm.2016.04.063 27215691

[29] Brooks MB: Penile prosthesis: a new device for corpus cavernosal dilation. *Urology.* 1989;34(4):225–6. 10.1016/0090-4295(89)90379-8 2800089

[30] Henry G Houghton L Culkin D: Comparison of a new length measurement technique for inflatable penile prosthesis implantation to standard techniques: outcomes and patient satisfaction. *J Sex Med.* 2011;8(9):2640–6. 10.1111/j.1743-6109.2011.02340.x 21679300

[31] Mulcahy JJ: Surgical management of penile prosthesis complications. *Int J Impot Res.* 2000;12 Suppl 4:S108–11. 10.1038/sj.ijir.3900587 11035396

[32] Moncada I Martínez-Salamanca JI Jara J: Inflatable penile prosthesis implantation without corporeal dilation: a cavernous tissue sparing technique. *J Urol.* 2010;183(3):1123–6. 10.1016/j.juro.2009.11.048 20092840

[33] Antonini G Busetto GM De Berardinis E: Minimally invasive infrapubic inflatable penile prosthesis implant for erectile dysfunction: evaluation of efficacy, satisfaction profile and complications. *Int J Impot Res.* 2016;28(1):4–8. 10.1038/ijir.2015.33 26657316 PMC4709472

[34] Sharma D Smith RP: Troubleshooting intraoperative complications of penile prosthesis placement. *Transl Androl Urol.* 2017;6(Suppl 5):S892–S97. 10.21037/tau.2017.07.13 29238668 PMC5715183

[35] Fishman IJ: Corporeal reconstruction procedures for complicated penile implants. *Urol Clin North Am.* 1989;16(1):73–90. 2916281

[36] Quinn AD Das S: Proximal extrusion of semirigid penile prosthesis. *Scand J Urol Nephrol.* 1989;23(3):239–40. 10.3109/00365598909180850 2799299

[37] Arrabal-Polo MÁ López-Carmona Pintado F González-Torres S: Transurethral extrusion of penile prosthesis. *Urol J.* 2013;10(3):941. 10.22037/uj.v10i3.2292 24078499

[38] Mulcahy JJ: Distal corporoplasty for lateral extrusion of penile prosthesis cylinders. *J Urol.* 1999;161(1):193–5. 10.1016/S0022-5347(01)62094-9 10037396

[39] Shaeer O: Management of distal extrusion of penile prosthesis: partial disassembly and tip reinforcement by double breasting or grafting. *J Sex Med.* 2008;5(5):1257–62. 10.1111/j.1743-6109.2008.00785.x 18331264

[40] Karpman E Sadeghi-Nejad H Henry G: Current opinions on alternative reservoir placement for inflatable penile prosthesis among members of the Sexual Medicine Society of North America. *J Sex Med.* 2013;10(8):2115–20. 10.1111/jsm.12203 23679798

[41] Henry G Hsiao W Karpman E: A guide for inflatable penile prosthesis reservoir placement: pertinent anatomical measurements of the retropubic space. *J Sex Med.* 2014;11(1):273–8. 10.1111/jsm.12361 24274160

[42] Stember DS Garber BB Perito PE: Outcomes of abdominal wall reservoir placement in inflatable penile prosthesis implantation: a safe and efficacious alternative to the space of Retzius. *J Sex Med.* 2014;11(2):605–12. 10.1111/jsm.12408 24286533

[43] Shelling RH Maxted WC: Major complications of silicone penile prosthesis: predisposing clinical situations. *Urology.* 1980;15(2):131–3. 10.1016/0090-4295(80)90403-3 7355536

[44] Wilson SK Delk JR 2nd: Prevention and treatment of complications of inflatable penile prosthesis surgery: a review article. *Arch Esp Urol.* 1996;49(3):306–11. 8702353

[45] Kaufman JJ Lindner A Raz S: Complications of penile prosthesis surgery for impotence. *J Urol.* 1982;128(6):1192–4. 10.1016/S0022-5347(17)53418-7 7154171

[46] Morgenstern JH Stein BS Kendall AR: Complications in patient with triple penile prosthesis. *Urology.* 1982;20(5):530–1. 10.1016/0090-4295(82)90129-7 7147532

[47] Carson CC Mulcahy JJ Govier FE: Efficacy, safety and patient satisfaction outcomes of the AMS 700CX inflatable penile prosthesis: results of a long-term multicenter study. AMS 700CX Study Group. *J Urol.* 2000;164(2):376–80. 10.1016/S0022-5347(05)67364-8 10893589

[48] Steege JF Stout AL Carson CC: Patient satisfaction in Scott and Small-Carrion penile implant recipients: a study of 52 patients. *Arch Sex Behav.* 1986;15(5):393–9. 10.1007/BF01543110 3789903

[49] Holloway FB Farah RN: Intermediate term assessment of the reliability, function and patient satisfaction with the AMS700 Ultrex penile prosthesis. *J Urol.* 1997;157(5):1687–91. 10.1016/S0022-5347(01)64835-3 9112506

[50] Rajpurkar A Dhabuwala CB: Comparison of satisfaction rates and erectile function in patients treated with sildenafil, intracavernous prostaglandin E1 and penile implant surgery for erectile dysfunction in urology practice. *J Urol.* 2003;170(1):159–63. 10.1097/01.ju.0000072524.82345.6d 12796670

[51] Tefilli MV Dubocq F Rajpurkar A: Assessment of psychosexual adjustment after insertion of inflatable penile prosthesis. *Urology.* 1998;52(6):1106–12. 10.1016/S0090-4295(98)00362-8 9836564

[52] Akin-Olugbade O Parker M Guhring P: Determinants of patient satisfaction following penile prosthesis surgery. *J Sex Med.* 2006;3(4):743–8. 10.1111/j.1743-6109.2006.00278.x 16839332

[53] Mulhall J Land S Parker M: The use of an erectogenic pharmacotherapy regimen following radical prostatectomy improves recovery of spontaneous erectile function. *J Sex Med.* 2005;2(4):532–40; discussion 540–2. 10.1111/j.1743-6109.2005.00081_1.x 16422848

[54] Katz D Bennett NE Stasi J: Chronology of erectile function in patients with early functional erections following radical prostatectomy. *J Sex Med.* 2010;7(2 Pt 1):803–9. 10.1111/j.1743-6109.2009.01516.x 19796019

[55] Müller A Parker M Waters BW: Penile rehabilitation following radical prostatectomy: predicting success. *J Sex Med.* 2009;6(10):2806–12. 10.1111/j.1743-6109.2009.01401.x 19732314

[56] Segal RL Camper SB Ma L: Prediction model for penile prosthesis implantation for erectile dysfunction management. *Curr Med Res Opin.* 2014;30(10):2131–7. 10.1185/03007995.2014.936188 24945719

