# Supplementary material for: Inflatable penile prosthesis in the radical prostatectomy patient: a review
Source: F1000Res. 2018 Jun 19;7:770. [Version 1] doi: 10.12688/f1000research.15241.1 (PMC6039953; doi:10.12688/f1000research.15241.1)
Supplement: Peer review history

# Peer review: Referee response for version 1

**DOI:** 10.5256/f1000research.16602.r35273

**Published:** 2018-07-06

**Authors:** Lawrence Jenkins

**Affiliations:** 1 Ohio State University, Columbus, OH, USA

Well put together manuscript reviewing the literature in penile prosthesis placement after radical prostatectomy. I commend the authors on their thorough summary. There are several points I would recommend clarified.

 1.       Page 2 paragraph 3 first sentence – “The initiating factor in the development…” This singular cause is debatable. There was a paper by Box et al. ^1^ that showed no difference with preservation of the APAs. I would recommend softening the tone.

 2.       Page 2 paragraph 4 – when discussing the potency rates I would recommend stating that those numbers include patients with and without the use of PDE5Is.

 3.       Page 2 – surgical considerations – 1 ^st^ paragraph, last sentence – spell check – Dilamezinsert is the name listed in the Cooper Surgical catalog.

 4.       Page 3 – 3 ^rd^ paragraph, prevention and management of perforation – Please clarify how the ‘U-type’ suture is used to prevent cylinder migration.

 5.       Page 3 satisfaction rates after penile prosthesis implantation, first sentence -  Check grammar, “of” can be removed as the 4 ^th^ word.

I have read this submission. I believe that I have an appropriate level of expertise to confirm that it is of an acceptable scientific standard.

## References

[1] BoxGNKaplanAGRodriguezESkareckyDWOsannKEFinleyDSAhleringTE: Sacrifice of accessory pudendal arteries in normally potent men during robot-assisted radical prostatectomy does not impact potency. *J Sex Med*.2010;7(1 Pt 1) : 10.1111/j.1743-6109.2009.01459.x 298-303 10.1111/j.1743-6109.2009.01459.x 19912507

# Peer review: Referee response for version 1

**DOI:** 10.5256/f1000research.16602.r35266

**Published:** 2018-06-27

**Authors:** Rafael E Carrion

**Affiliations:** 1 Department of Urology, Morsani College of Medicine, University of South Florida, Tampa, FL, USA

The authors provide a review manuscript highlighting the role of a specific surgical modality in the realm of prostate cancer survivorship.  They initially start by providing a concise review of ED after RP.

 Under "Surgical Considerations," they begin to discuss specific steps of the penile implantation surgical process.  I felt they provided a good review on the separate domains of penile implant surgery.  However, I would avoid specific surgeon bias specific steps such as the first sentence under "Technique of corporal measurement," where the author's state, "After corporotomy, PDS 2-0 stay sutures..."  This is NOT a surgical techniques manuscript, it is a review of the topic at hand.

I have read this submission. I believe that I have an appropriate level of expertise to confirm that it is of an acceptable scientific standard.
